# The Single-tooth Implant Treatment of Congenitally Missing Maxillary Lateral Incisors Using Angled Abutments: A Clinical Report

**Published:** 2009

**Authors:** Suleyman Hakan Tuna, Filiz Keyf, Gurel Pekkan

**Affiliations:** * Assistant Professor, Department of Prosthodontics, School of Dentistry, Suleyman Demirel University, Isparta, Turkey; ** Professor, Department of Prosthodontics, School of Dentistry, Ankara, Dr Hacettepe University, Turkey; *** Assistant Professor, Department of Dentistry, Dumlupinar University, Kutahya, Turkey

**Keywords:** Dental abutment, Dental implants, Emergence profile, Incisor, Maxilla

## Abstract

The maxillary lateral incisor is the second most common congenitally absent tooth. There are several treatment options for replacing the missing maxillary lateral incisor, including canine substitution, tooth-supported restoration, or single-tooth implant. Dental implants are an appropriate treatment option for replacing missing maxillary lateral incisor teeth in adolescents when their dental and skeletal development is complete. This case report presents the treatment of a patient with congenitally missing maxillary lateral incisors using dental implants with angled abutments.

## Introduction

Tooth loss in the anterior region is commonly the result of a traumatic injury or a congenital anomaly. The most common congenitally missing anterior tooth is the maxillary lateral incisor. Several options are available for the replacement of congenitally missing maxillary lateral incisor tooth.^1^–^4^ These include removable dental prostheses, conventional fixed dental prostheses (FDPs), resin-bonded FDPs, orthodontic repositioning of canines to close the edentulous space, and single-tooth implant.^1^,^4^,^5^ Removable dental prosthesis is often used as a transitional restoration for a maxillary lateral incisor tooth.^5^ The traditional treatment for an edentulous space in maxillary lateral incisor area is a conventional three-unit or cantilever FDP. A major shortcoming of these alternatives is the significant tooth reduction of the abutments. Subgingival margins are required in esthetic situations, but these are associated with increased gingival inflammation.^6^ While some clinicians may suggest that a resin-bonded prosthesis is a viable option, clinical experience has shown that these resin-bonded pontics do not have a good long-term success rate if the teeth are not prepared aggressively enough for mechanical retention. Debonding rates of 25-31% have been reported for these restorations.^7^,^8^ In cases where the occlusion and esthetics of the canine in the lateral position are acceptable, closure of the lateral space by the mesially positioned canine may be the simplest alternative treatment option.^1^,^3^ Dental implant is an appropriate treatment option for replacing missing maxillary lateral incisor tooth in adolescents when their dental and skeletal development is complete.^9^–^11^ For males, completion of facial growth, which of-ten corresponds to general growth, may not occur until the age of 21 years; in young women, growth may be completed by age 15.^3^,^9^ If growth is complete, dental implants can be placed as soon as the edentulous space has been created and the tissues have stabilized following orthodontic treatment. The cervical esthetics of a single implant crown must accommodate a round diameter implant and balance hygiene and esthetic parameters.^12^ In the anterior maxilla, the placement of an implant in a prosthetically ideal position is often not possible because of the lack of sufficient bone, vertically or horizontally.^13^ Because of esthetic or spatial needs, angled abutments are often needed after placement of dental implants in the esthetic zone.^14^,^15^ The preservation of soft tissues and regeneration of inter-dental papillae are critical for the esthetic success of single-implant-supported crowns.^16^,^17^ This case report presents the single-tooth implant treatment of a patient with congenitally missing maxillary lateral incisors using angled abutments.

## Case Report

**Figure 1:**
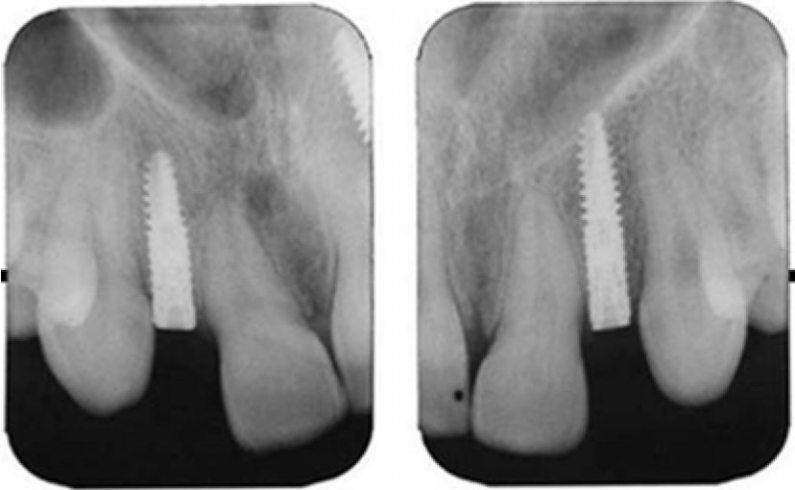
Periapical radiographs after osseointegration

An 18-year-old female patient with congenitally missing maxillary lateral incisors referred to the Department of Proshodontics, Hacettepe University. Her medical and dental history was evaluated. Dental history revealed that she had received orthodontic treatment for obtaining proper space for prosthetic restoration of her missing lateral incisors. She was wearing Hawley retainer. Periapical and panoramic radiographs and preliminary impression were taken for diagnostic evaluation. A decision was made for dental implant treatment. A maxillary surgical stent was prepared for the correct implant position. The formal surgical procedure recommended by the manufacturer of the dental implant system (XiVE®, DENTSPLY Friadent GmbH, Mannheim, Germany) was followed. At the first stage of surgery, a large fossa and horizontal bone deficiency was observed on the labial alveolar bone. To overcome bone deficiency, small diameter implants were selected and placed with labial angulation to avoid labial fenestration (a 3 × 15 mm XiVE S CELLplus^TM^, DENTSPLY Friadent GmbH For the left maxillary lateral incisor and a 3 × 13 mm XiVE S CELLplus^TM^, DENTSPLY Friadent GmbH for the right maxillary lateral incisor) (Figure 1). The implant shoulder was placed to the 2 mm apical part of the cemento-enamel junction of the neighboring teeth, with the aid of surgical stent. Thereafter, submerged cover screws were fastened, and the flap was precisely repositioned and sutured free of tension. During the healing period, the patient wore an acrylic provisional removable dental prosthesis relieved in the implantation sides. After three months, at the second stage surgery, cover screws were removed, and gingiva formers were located.

**Figure 2:**
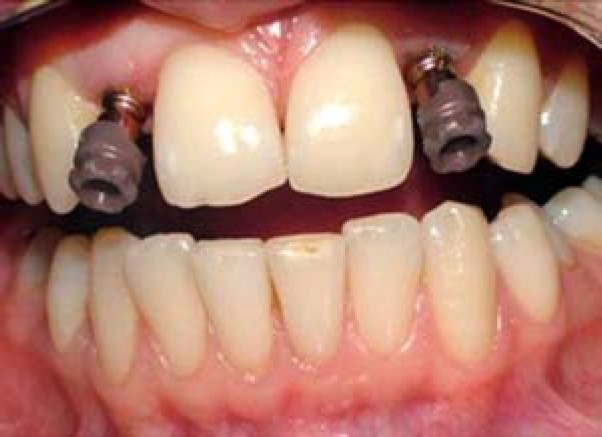
Transfer copings with transfer caps in place before final impression making.

Thus, gingival formation was delayed in order to minimize the soft tissue recession. At the fourth month, gingival formers were unscrewed and final impression of the maxillary arch was made using vinyl polysiloxane impression material (Elite HD, Zhermack SpA, Badia Polesine (Rovigo), Italy) while the transfer copings and caps were in place (Figure 2).

**Figure 3:**
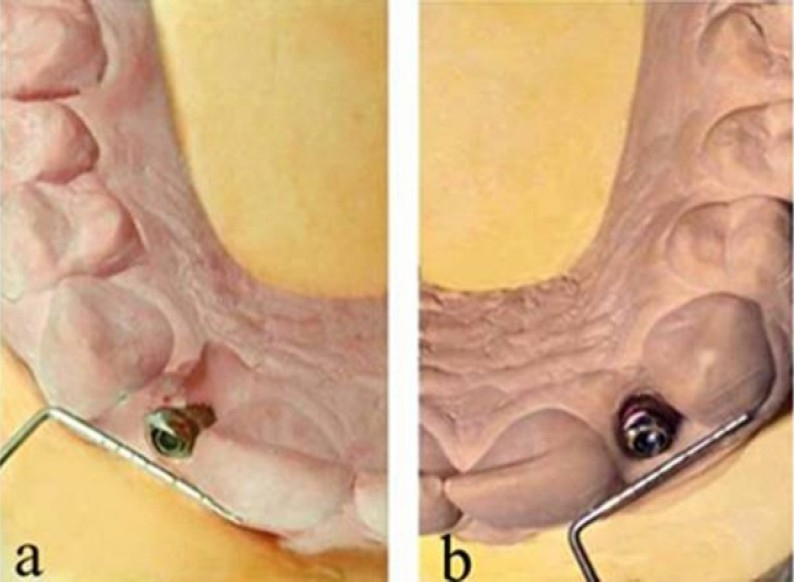
Probing instrument showing the orofacial positioning of the angled abutments after preparation a. left side, b. right side.

The abutment analogs were secured in their places in the impression and the cast was poured in type IV hard plaster (Begostone plus, BEGO Bremer Goldschlagerei Wilh. Herbst GmbH & Co. KG, Bremen, Germany). Abutment selection was performed in the patient’s mouth and on the definitive cast. Standard abutments were excessively in labial position that would not allow proper crown placement. Angled abutments (Dentsply Friadent GmbH) were chosen due to the axial-position problem (orofacial direction) of the implants. However, even angled abutments were insufficient to eliminate excessive labial emergence; therefore, they were prepared using laboratory implant analogs and analog holder to achieve sufficient emergent profile and to optimize place for the ceramic crowns (Figure 3).

**Figure 4:**
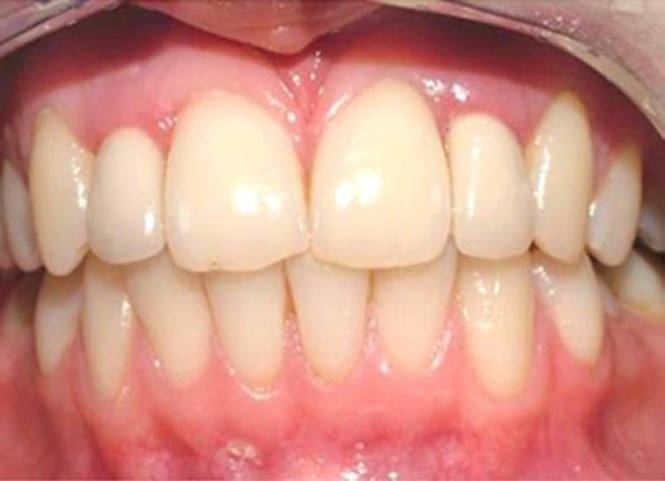
Close-up view of the final restorations after cementation.

Temporary crowns were prepared on the master cast using temporary restoration material (Dentalon Plus, Heraeus Kulzer GmbH & Co. KG, Hanau, Germany). Abutments were attached to the implants, screwed onto them and tightened to 35 Nm using ratchet and ratchet hex driver. Temporary crowns were cemented using temporary cement (PreVISION CEM, Heraeus Kulzer GmbH & Co. KG) on abutments for two weeks aiding also for maintaining the tissue form while the permanent crowns were fabricated. Metal ceramic crowns were finished and tried in their places.

Radiographs were taken at baseline and after 6, 12 and 24 months in order to control the bone level around implants (Figure 6).

**Figure 5:**
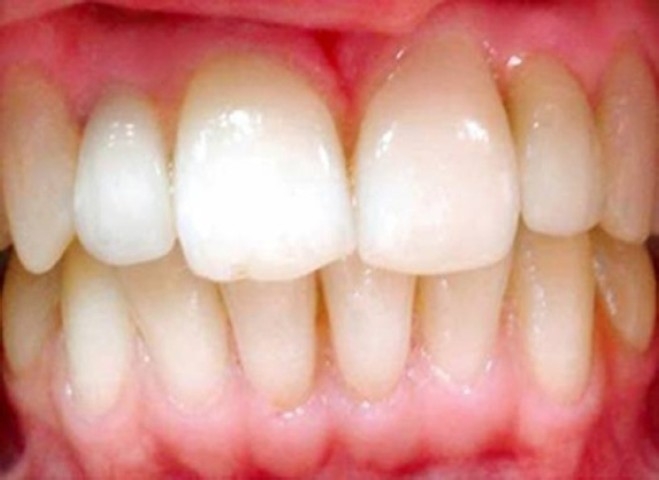
Close-up view after two years. Note the interdental papilla levels.

The patient was informed on oral hygiene and instructed in the specific care for her new restorations including tooth brushing and flossing. Follow ups were done at monthly intervals for three months, and then once every six months for two years (Figure 5)

**Figure 6:**
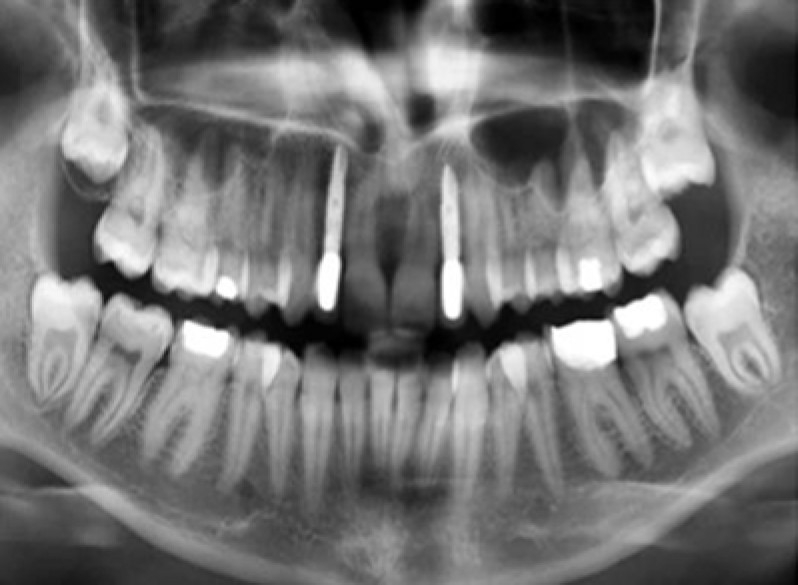
Panoramic radiograph taken after two years.

## Discussion

Long-term studies have shown that the success rate of the osseo-integrated implants reach almost 100%.^2^,^11^,^18^ Although, the use of dental implants in the esthetic zone is well documented in the literature, placing dental implants in the anterior maxillary area is considered to be the ultimate challenge for many dentists.^2^–^6^,^9^ In some patients, the space across the alveolar crest is too narrow to permit placement of an implant.^12^ Occasionally, the root apices of the adjacent central incisor and canine are in close proximity.^1^,^3^ In other cases, ridge thickness may be inadequate, requiring soft tissue or bone augmentation.^9^,^17^ The placement of implants in a correct three-dimensional position is one of the keys to an esthetic treatment outcome.^5^,^9^ Conventional radiography, computed tomography, diagnostic bone probing, and bone thickness measurement with gauge are invaluable methods to evaluate the alveolar bone, three-dimensionally in the esthetic zone before surgery.^2^,^5^,^9^ For this patient, panoramic and periapical radiographs were used in radiographic evaluation of alveolar bone in the maxillary lateral incisor area before surgery. However, they did not give enough information and failed to detect the horizontal bone deficiencies seen during surgery. These deficiencies were not obvious before surgery because of the presence of soft tissue bulk over them. Surgical stents are used for proper positioning of the implants during surgery. Recently, surgical guides fabricated using stereo-lithographic techniques or computer assisted design and computer assisted manufacturing (CAD/CAM) systems aid more precise and exact positioning of the implants during surgery.^19^,^20^ Surgical navigation techniques may also be used in challenging situations.^21^ The quantity and quality of alveolar bone as well as soft tissues around the implant must be assessed carefully before considering implant placement especially in the esthetic zone.^3^ Horizontal and vertical bone deficiencies cause esthetic complications. To accommodate a standard implant in the maxillary lateral incisor area, there should be a minimum of 10 mm of inciso-gingival bone and a minimum of 6.0 mm of facial-lingual bone. Adequate space for the implant is also required between the adjacent roots. One to 2 mm of space is necessary between the implant and the adjacent roots.^22^ Fortunately, the cervical diameter of maxillary lateral incisor is the most similar to that of the implant. In cases where there is insufficient alveolar bone for implant placement, ridge augmentation may be necessary.^3^ However, using narrow-diameter implants in these cases seems to be a treatment option as predictable as using standard-diameter implants.^12^,^23^ In this case, alveolar bone was available in maxillary lateral incisor areas in the mesiodistal and coronoapical dimen-sion; however, there was deficiency in orofacial dimension. The patient was refused to have bone augmentation procedures using either autogenic or synthetic bone grafts because of financial and patient related factors. Therefore, implants with 3 mm diameter were used to compensate for horizontal alveolar bone deficiency. However, to avoid labial fenestration, the implants had to be placed off axis in labial direction. The relationship of the position between the implant and the proposed restoration should be based on the position of the implant shoulder, since it will influence the final hard and soft tissue response.^24^ The malposition of the implant shoulder in the coronoapical direction causes soft tissue recession.^9^ In this case, location of the implant shoulders was in coronoapical and mesiodistal dimension in comfort zone. However, in the orofacial dimension the implant shoulders were in danger zone.^9^ The angulation of implants in labial direction was compensated using angled abutments that were prepared for better emergence profile of the ceramic crowns. Many authors have also concluded that angled abutments may be considered a suitable restorative option when implants are not placed in ideal axial positions.^14^,^25^,^26^ Nevertheless, forces applied off axis may be expected to overload the bone surrounding single-tooth implants, as shown by Papavasiliou et al^27^ using finite element analysis. Hence, the segmental osteotomy may provide an alternative treatment to reposition the severely malposed implants.^28^ To optimize esthetic treatment outcomes, the use of provisional restora-tions with adequate emergence profiles is recommended to guide and shape the peri-implant tissue and to prevent collapse before definitive restoration.^13^,^24^ After placing immediate provisional restoration, the soft tissue recession is expected much more than delayed provisionalization. Therefore, in current case, provisionalization was delayed.^9^ However, it has reported that there is no difference between delayed or immediate provisionalization and papilla scores.^29^ Stock implant abutments are traditionally made of gold alloy or titanium. Recently, stock or custom zirconia or alumina abutments made with CAD/CAM technologies have increasingly been used.^9^,^30^ It must be kept in mind that when the abutment retaining screw access and angle correction are taken into account, the resulting abutments are often left with very thin sections. Therefore, ceramic abutments are at risk of fracture due to these dimensions. In this case, although metal abutment and metal-ceramic crowns were used, there was no submucosal reflection of the metal abutment. Dental implants can be restored with cemented or screw-retained FDPs. In most esthetic areas, the implant shoulder is located subgingivally, resulting in a deep interproximal margin. This shoulder location makes seating of the restoration and removal of cement difficult. Therefore, screw retained restorations are mostly preferred in these cases. But, in the present case, because of the angulation of the implants, cemented restorations had to be chosen, although off axis implant placement can sometimes be compensated with angled abutments that still allow screw retention.^14^ Besides, after preparation of the angled abutments, the retention areas of the crowns were significantly reduced. For this reason, crowns were luted with adhesive resin cement. Cement remnants were removed easily because the implant shoulders were not deeply located.

## Conclusion

The dental implant treatment of a patient with congenitally missing maxillary lateral incisors that had horizontal alveolar bone deficiency was performed using narrow diameter implants and angled abutments. At the 2-year follow-up, it was concluded that treatment using angled abutments were satisfactory for the patient’s esthetic expectations. Interdental papilla levels were increased gradually and improved natural appearance.
